# Single-port flexible endoscopic enterolysis under the guidance of electromagnetic navigation in a hypochlorite-induced abdominal adhesions pig model

**DOI:** 10.3389/fsurg.2026.1720046

**Published:** 2026-02-18

**Authors:** Airong Tang, Wei Shen, Yufeng Xu, Cuiyun Ma, Zhenfeng Xu, Yan Liu

**Affiliations:** 1 Department of Gastroenterology, The 5th Medical Center of PLA General Hospital, Beijing, China; 2 Beijing Huahang Radio Measurement Institute, Beijing, China

**Keywords:** adhesiolysis, electromagnetic navigation, endoscopy, laparoscopy, small bowel obstruction

## Abstract

**Purpose:**

Small bowel obstruction (SBO) usually resolves with medical therapy but requires surgery, preferentially under the guidance of laparoscopy. We performed single-port flexible endoscopic enterolysis for abdominal adhesions under the guidance of dual-channel electromagnetic navigation technology and evaluated the feasibility and safety of this method in a pig model.

**Method:**

Hypochlorite-induced abdominal adhesions was induced in 8 pigs. A long gastric tube with a magnetic navigation wire (sensor 1) was inserted into the distal end of the small intestine. The electromagnetic signal emitter was activated, and a second magnetic navigation wire (sensor 2) was continuously adjusted on the abdominal surface to obtain the two coordinates closest to the incised skin. The trocar was placed, and pneumoperitoneum was established. An endoscope with a second wire was placed through the trocar, and diagnostic intraperitoneal endoscopy was performed. The fibrotic bands and peritoneal congestion were observed, and the fibrous bands were dissected with a Dual knife.

**Results:**

Abdominal adhesions model was evaluated. One case experienced intraoperative bleeding because the small mesenteric artery was damaged. Both the peritoneum and operating space were sufficiently visible in all cases except one, in which intestinal inflation significantly affected visibility. Seven of 8 (87.5%) of fibrous bands were successfully dissected. The total procedure duration was 48 (35–60) mins. Compared with that in the hypochlorite-induced abdominal adhesions model, weight gain was 6 (4–9) kg.

**Conclusion:**

Flexible endoscopy adhesiolysis under the guidance of electromagnetic navigation for abdominal adhesions is comparatively safe and feasible.

## Introduction

Small bowel obstruction (SBO) is a common yet often complex for the general surgeon to treat. The most common aetiology is adhesion due to peritoneal trauma and subsequent inflammation (1). Although most patients remain asymptomatic after the development of postoperative adhesions, 20% of all surgical emergencies are due to adhesion-related bowel obstruction (2). Abdominal adhesions begin forming within hours after abdominal surgery, accounting for 60% of intestinal obstructions (3). Although various pharmacological agents and surgical strategies have been developed to prevent the formation of adhesions, none have shown impressive effects (4). Therefore, advances in the management of SBO remain difficult to implement. SBO usually resolves with surgery, preferentially under the guidance of laparoscopy, to prevent total obstruction, bowel perforation, severe ischaemia, or clinical deterioration (1). The rates of recurrence are high because the surgery itself can cause new adhesions to form, with approximately 10% to 30% of patients requiring another laparotomy (5). Owing to the burden of SBO on patients and the healthcare system, identifying potentially modifiable risk factors for postoperative SBO is important. Putative risk factors include age, previous abdominal surgery, previous history of SBO, peritonitis, emergency surgery, and surgery type (6). At present, single-port laparoscopy, natural orifice transluminal endoscopic surgery (NOTES) and robotic single-port laparoscopy are considered new clinical surgical strategies that are less invasive and result in faster postoperative recovery (7, 8). However, one of these surgical strategies has several limitations, such as limited manoeuvrability of the instruments due to limited space, interferences between the instruments during single-port laparoscopy, spatial disorientation in natural orifice transluminal endoscopic surgery (NOTES), increased cost and long training time, particularly for robotic single-port laparoscopy.

There are minimally invasive surgical techniques in every surgical discipline. Opting for a minimally invasive approach whenever feasible reduces the risk of peritoneal damage, thereby minimizing the risk of adhesion formation. Several studies have shown that SBO patients treated via the laparoscopic approach experience fewer complications, faster recovery of bowel function and a lower incidence of abdominal adhesion formation (9). Owing to the development of NOTES, the single-port laparoscopic technique has been widely accepted by surgeons. However, single-port laparoscopy requires the use of rigid scopes and instruments, which makes the detection and treatment of abdominal adhesions difficult. The single-port laparoscopic technique has not been widely used in intra-abdominal adhesiolysis. In recent years, laparoscopy and endoscopy cooperative surgery (LECS) was developed (10). LECS has the advantages of both flexible endoscopy and rigid laparoscopy to achieve super minimally invasive surgery (11–13).

Compared with those of rigid laparoscopy, the prominent advantages of flexible endoscopy include clear visualization of all corners of the abdominal cavity through the flip endoscopic end, and some equipment, such as electric knives, biopsy forceps, and snare, and transmission of images to the operation field for observation and treatment through the biopsy channel. However, the procedure is more difficult than traditional laparoscopic surgeries because of its technical challenges, including crowding of the laparoscope and instruments around the umbilicus and the loss of triangulation between the two instruments in the operative field, leading to spatial disorientation. Therefore, flexible endoscopy is not feasible for the exploration of lesions deep within the abdominal cavity.

Electromagnetic navigation is a new type of surgical navigation technology that has been introduced in recent years. Electromagnetic tracking devices have been used to locate lesions during neurosurgery, urology, endonasal procedures, and cardiac procedures (14). Although research has revealed that multiple electromagnetic sensors can quickly locate lesions (15), they are not well suited for use in the moving anatomy of the intraperitoneal compartment. To overcome the difficulties associated with real-time intraoperative localization during surgery, we performed a single-port flexible endoscopic surgical method to treat abdominal adhesions under the guidance of dual-channel electromagnetic navigation technology and evaluated the feasibility and safety of this method in a pig model.

## Methods

### Establishment of a hypochlorite-induced adhesive intestinal obstruction model

The study was approved by the Animal Ethics Center of PLA General Hospital. Eight pigs weighing 20–28 kg of either sex were raised by the Animal Center of PLA General Hospital. All the animals were fed commercial semisolid swine feed and housed in groups independently during a 7-day acclimatization period. Eight pigs were weighed and sedated via an intramuscular injection of a tiletamine/zolazepam (4 mg/kg) mixture. NaClO solution (Sigma) was given intraperitoneally. Moreover, 20 mL/kg of B.W. (∼ 400 mL/pig) sterilized normal saline containing 0.2% (v/v, 30 mM) chemical irritant was used (16). All pigs were subsequently housed in the animal facility as described above. Food intake and gastrointestinal symptoms were observed daily. The final weight was recorded on the fourteenth day, after which adhesiolysis was performed. At the end of the study, the animals were euthanized with an overdose of potassium chloride under deep anesthesia using a mixture of intramuscular ketamine (6 mg/kg), acepromazine (0.5 mg/kg).

### Operation method

#### Early-stage preparations

**Figure 1:**
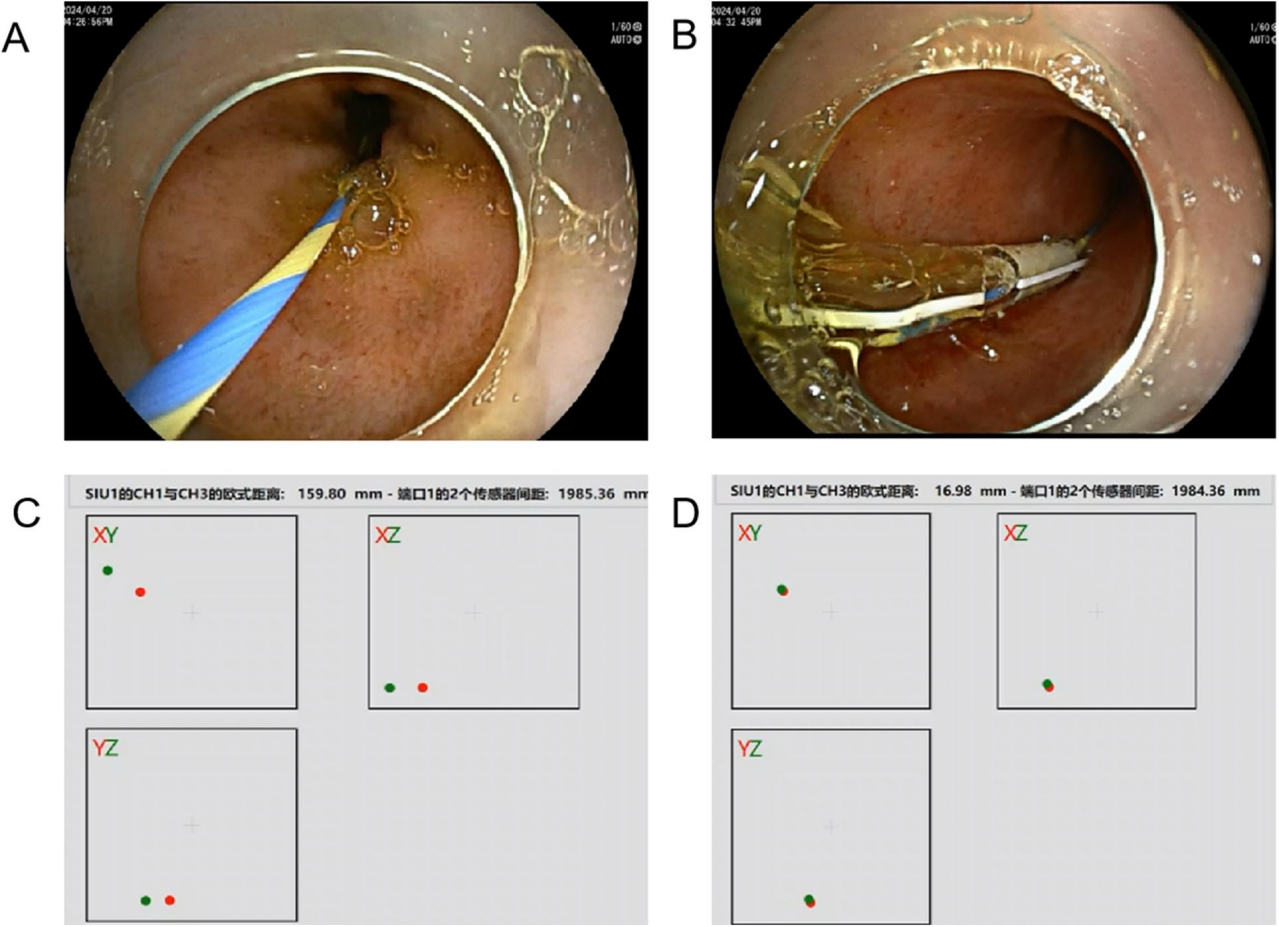
Placement of the guide wire **(A)**; insertion of a gastric tube along the guide wire under direct endoscopic visualization **(B)**; the spatial distance between the two guidewires after entering the abdominal cavity **(C,D)**.

A senior endoscopist performed the procedure at the animal centre. During the operation,

intubation inhalation anesthesia was induced by the veterinary team. With the animals in the supine position, The guidewire (0.035 inch, Micro-Tech, Nanjing, China) is advanced to the small intestine through the endoscopic biopsy channel (GIF TYPE H260, Olympus Corporation, Tokyo, Japan),then, with retaining the guidewire and withdrawing the endoscope, and then under direct endoscopic visualization, a 5.3-mm diameter long gastric tube measuring 110 cm in length (Yikai Medical Device Corporation, Jiangsu, China) was inserted along the guidewire to the distal end of the small intestine as far as possible and fixed for decompression of the intestinal contents (Figures 1A,B).

#### Endoscopic enterolysis

Step 1: When no liquid was drained from the gastric tube, a 2-mm diameter magnetic navigation wire (sensor 1) measuring 170 cm in length (AK47-AMT170C, Beijing, China) was inserted along the gastric tube to the distal end of the gastric tube. The depth of wire insertion was estimated according to the scale of the gastric tube. Moreover, the electromagnetic navigation signal emitter was activated, and the coordinates of the two sensors on the monitoring screen were displayed. Distance

data were recorded using Euclidean distance, which represents the actual distance between two points in the multidimensional space. At this time, a second magnetic navigation wire (sensor 2), also 2 mm in diameter but 320 cm in length (AK47-AMT320C, Beijing, China), was continuously adjusted on the abdominal surface so that the two coordinates were closest. The data were approved by both the doctor and engineer and collected three times consecutively. The search time and distance were recorded.

**Figure 2:**
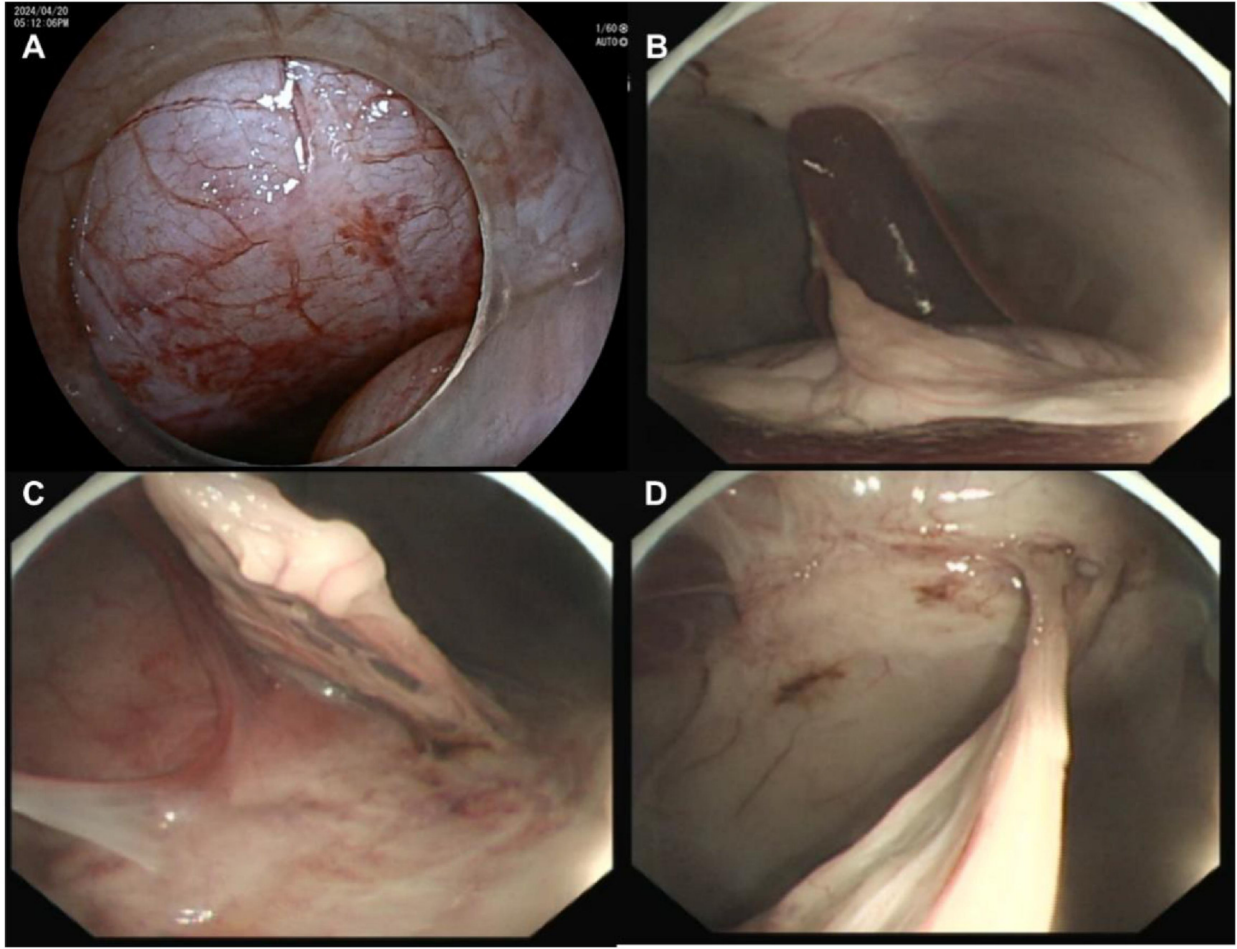
Peritoneal injury **(A)** and abdominal adhesions **(B–D)**.

**Figure 3:**
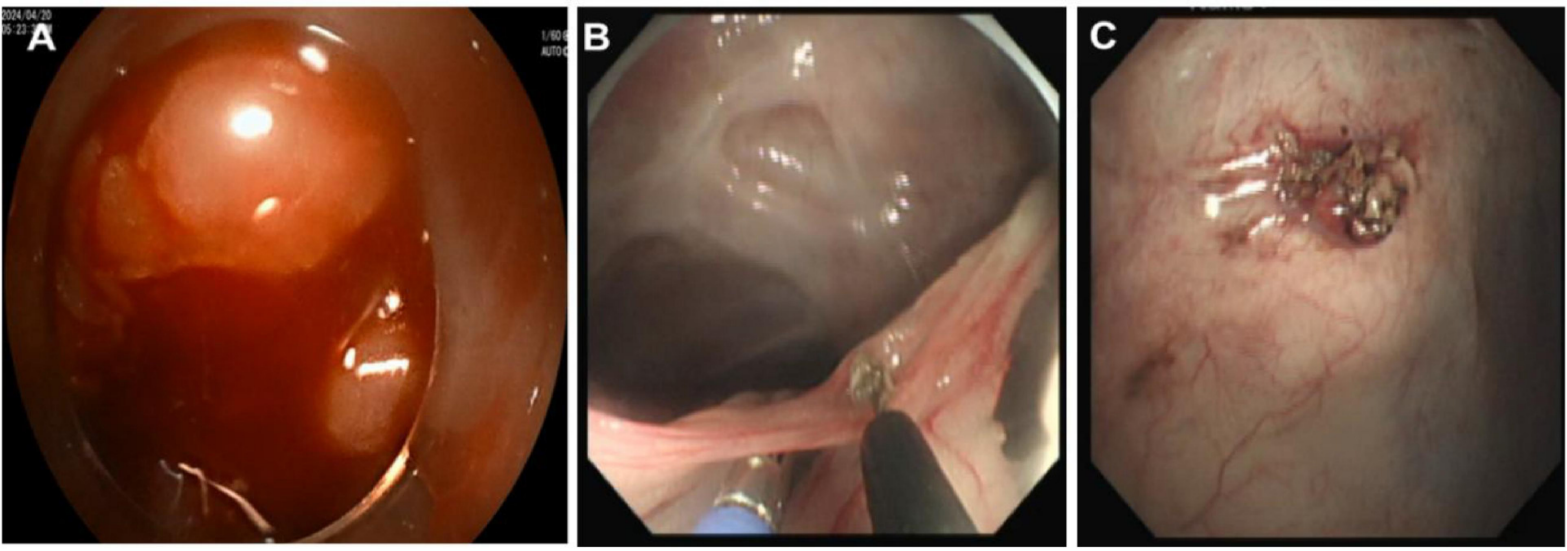
Intraoperative bleeding **(A)** and severing adhered greater omentum **(B, C)**.

Step 2: A vertical skin incision of 2–3 cm was made at the point closest to sensor 1. Second, a trocar (Hangzhou Kangyuan Medical Instrument Corporation, Hangzhou, China) was placed. This trocar also has an extra tube that allows CO2 infiltration. Pneumoperitoneum was subsequently established by adjusting the pressure to 8 mm Hg to maintain good visibility in the abdominal cavity. Third, the second new magnetic navigation wire was inserted into the end of a dual-channel endoscope (GIF TYPE 2TQ260M, Olympus Corporation, Tokyo, Japan). The endoscope was placed through a trocar, and diagnostic intraperitoneal endoscopy was performed beginning approximately 5 cm around the first electromagnetic signal (Figures 1C,D). The whole abdominal cavity was observed first to determine whether there was obvious bleeding, gastrointestinal perforation or solid organ trauma. To observe local situations, the key is abdominal cavity adhesion. We attempted to examine adhesions among the intestines using biopsy forceps by gently lifting the intestinal wall. If an injury was observed, endoscopic treatment was attempted. The mesojejunum was tightly adhered to the parietal peritoneum. A fibrotic band, adhered greater omentum and exhibiting peritoneal congestion were also observed (Figure 2). Fourth, the second wire was withdrawn, and fibrous band and adhered greater omentum were dissected using a Dual knife (KD-650 L, Olympus Corporation, Tokyo, Japan) and biopsy forceps (Anrui Medical Device Co. Hangzhou, China) to pull (Figures 3B,C). The search time and distance were recorded.

Step 3: After the operation was completed, the operative field was inspected to ensure that no perforation or haemorrhage remained. In the end, the pneumoperitoneum was aspirated via the endoscope. The fluid from the abdominal cavity and the intestine was collected for bacterial cultivation. The incision was sutured layer by layer. Every operation time was recorded.

### Follow-up

After the operation, all animals were denied food for 24 h. Amoxicillin (0.5 g) was subsequently given orally twice daily for the next 5 days. All the animals were carefully observed, and their general conditions, behaviour, and eating habits were recorded. The animals experienced an uneventful recovery during the first postoperative week, and no complications were observed. After the 14 days of observation, all the animals were euthanized.

### Outcome measurements

The primary endpoint was 14-day survival. The humane end point before the schedule was preliminarily determined when any of the following conditions were met: weight loss ≥20% within 1 week, fever >40°C for 1 week, or anorexia for 3 days or massive bleeding. An adhesion intestinal obstruction model was evaluated. The secondary endpoints were the rate of successful detection and dissection of the ligament and the occurrence of port site complications (including seroma formation, surgical site infection and wound dehiscence).

### Statistical analysis

Categorical data are expressed as frequencies with percentages, and continuous data are expressed as medians with interquartile ranges. No inferential statistical testing was performed.

## Results

### Surgical outcomes

**Figure 4:**
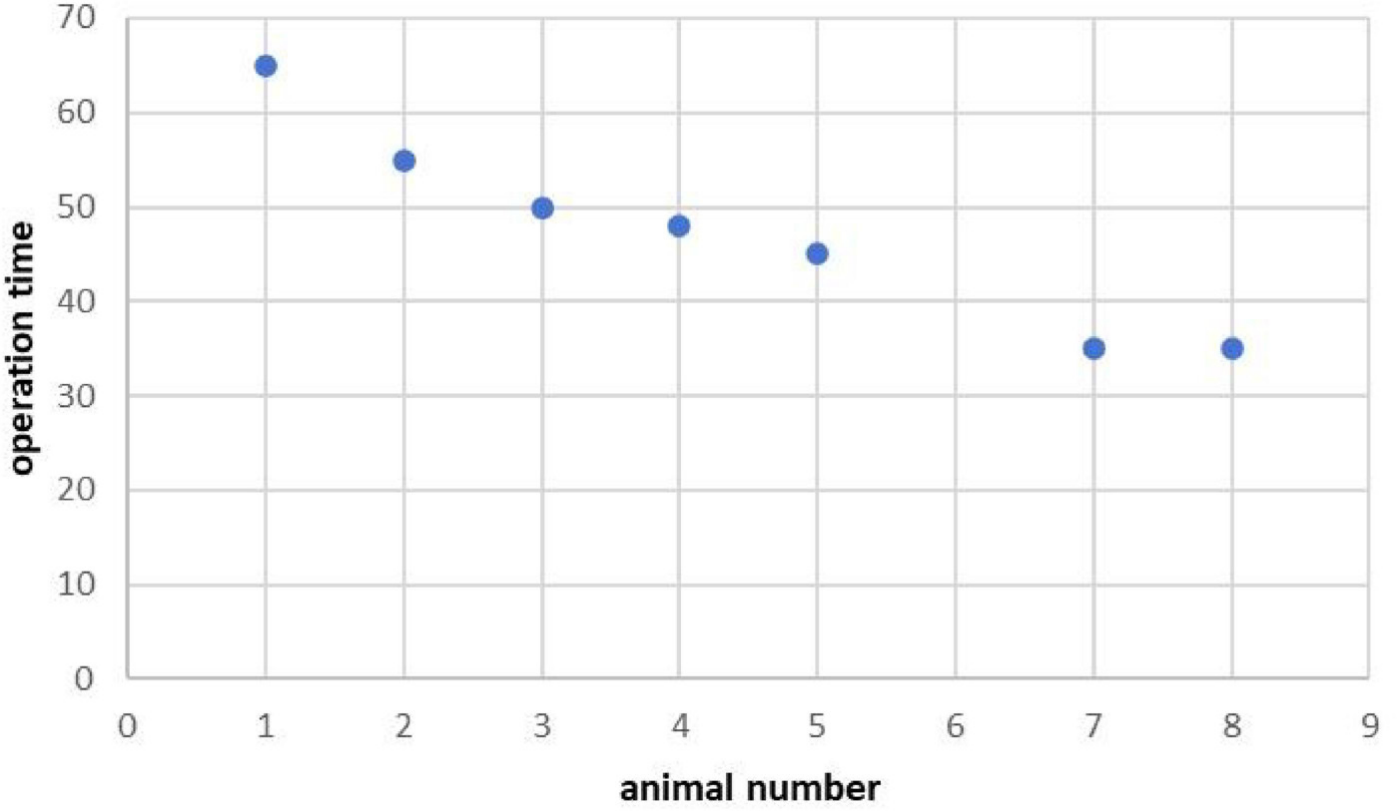
Operation time (mins).

First, the abdominal adhesions model was evaluated (Table 1). It was easy to obtain satisfactory visibility of the operating space despite intestinal inflation significantly affecting vision in one case. Some organs, such as the liver, gallbladder, spleen, and diaphragm, were clearly identified. The abdominal wall structures were clearly visualized via a flipping endoscope. No significant organ injury was found. We attempted to examine adhesions within the intestines using biopsy forceps and gently lifting the intestinal wall. However, the exploration of the small intestine is difficult, and the mesentery or intestinal wall often blocks the end of the endoscope without resolution of the pneumoperitoneum. The greater omentum was found tightly adhered to the parietal peritoneum. A fibrotic band compressing the intestine and varying degrees of peritoneal congestion were also observed. Simple intestinal-abdominal wall adhesion was the main type of adhesion. Moreover, 14-day survival was observed in all cases without any instances of the humane end point. Seven of 8 fibrous bands (87.5%) were successfully dissected. The duration of the procedure at each stage and the distance were recorded (Table 2). The durations of finding the closest location before incising the skin and after incising the skin were 3 (2–4) mins and 6 (5–8) mins, respectively. The time to find the closest distance between the two wires after the skin was incised was significantly longer than that before the skin was incised. The total procedure duration was 48 (35–60) mins, and no incision-related complications occurred. The time needed for electromagnetic localization was 21% (16%–27%) of the total operation time. Owing to an increasing number of operations, the operation time tended to decrease (Figure 4).

**Table 1:** Assessment of intestinal obstruction model.

Animal number	Intestine-abdominal wall adhesion		Intestinal adhesion	Intestine dilatation	operating space	Organ injury	peritoneum is visible
	Extensive adhesion	Simple adhesion					
P1	+	–	–	+	Poor	–	+
P2	–	+	–	–	Well	–	+
P3	–	+	–	–	Well	–	+
P4	–	+	–	–	Well	–	+
P5	–	+	–	–	Well	–	+
P6	–	–	–	–	–	–	–
P7	–	+	–	+	Well	–	+
P8	–	+	–	–	Well	–	+

**Table 2:** Procedural outcome of operation.

Animal number	Step1		Step2		Proximity distance between sensor 1 and 2 (mm)	Find Adhesions or mucosal damage	Operation time (min)
	Time-consuming on the closest distance (min)	Euclidean distance between sensor 1 and 2 (mm)	Time-consuming on the closest distance (min)	Endoscopic forward distance (mm)			
P1	4	30 (30–45)	8	50	4 (3–5)	+	60
P2	3	25 (25–30)	6	40	5 (4–5)	+	55
P3	2	35 (25–40)	6	50	4 (3–4)	+	50
P4	3	32 (30–40)	7	50	3 (3–4)	+	48
P5	4	33 (30–45)	8	60	5 (4–5)	+	45
P6	2	35 (33–46)	5	–	–	–	–
P7	2	32 (29–43)	6	60	4 (3–4)	+	35
P8	3	30 (29–35)	6	50	3 (3–4)	+	35

Step1: before skin incision.

Step2: after skin incision.

Continuous data are expressed as median.

Operative complications were recorded (Table 3). One case experienced intraoperative bleeding because the small mesenteric artery was damaged, and endoscopic haemostasis failed (Figure 3A). In another case, intraoperative bleeding was successfully resolved with haemostasis via endoscopic electrocoagulation. Therefore, the operation was terminated. One animal with multiple adhesions sustained an intestinal electrocautery injury and underwent intestinal expansion, resulting in poor peritoneal visibility and accidental injury. No intestinal perforation occurred. The exploration of adhesions deep within the intestines is restricted by equipment and technology.

**Table 3:** Operative complication.

Animal number	Hemorrhage	Intestinal injury	Intraperitoneal abscess	Incision infection	Adhesion recurrence
P1	–	+	–	–	–
P2	–	–	–	–	–
P3	–	–	–	–	–
P4	–	–	–	–	–
P5	–	–	–	–	–
P6	+	–	–	–	–
P7	–	–	–	–	–
P8	+	+	–	–	–

### Long-term survival

After being given a NaClO solution, the animals ate less and lost weight. After the operation, more food was eaten, and the weight increased. Normal activity 24 h after the procedure. Compared with that in the hypochlorite-induced abdominal adhesions model, the weight gain was 6 (4–9) kg. The original weight, seventh day after the addition of NaClO solution and weight changes are shown in Table 4. None of the animals required analgesics in the postoperative period. All pigs were ultimately euthanized after 14-day post-operation.

**Table 4:** Weight change (kg).

Weight(kg)	Animal number							
	1	2	3	4	5	6	7	8
Original weight	24.5	23.6	26.0	25.5	25.0	25.5	24.6	25.6
After giving NaClO	20.0	22.0	24.0	23.0	23.0	24.0	23.0	22.0
Final weight	29.0	30.0	30.0	27.0	28.0	–	29.0	30.0
Weight change	9.0	8.0	6.0	4.0	5.0	–	6.0	8.0

Weight change = weight (final weight- after giving NaClO).

### Infectious evaluation

No abdominal abscess or incisional infection was found. However, 7 cases were positive for *Escherichia coli*. No clinical signs of infection, such as fever or abdominalgia, were observed.

## Discussion

Adhesive small bowel obstruction (ASBO) is one of the leading causes of surgical emergencies worldwide and requires adhesiolysis surgery. Conventional open surgery has been the standard therapy for this disease, but the disadvantages of open surgery include increased postoperative pain, prolonged intestinal paresis, wound infection, and ventral incisional hernia, and poor cosmesis have limited its clinical application (17). Furthermore, operations carry the risk of causing more adhesions. Abdominal exploration through lapa­rotomy has been the preferred therapy for SBO in place of open surgery for simple intestinal adhesion. However, laparoscopic adhesiolysis for ASBO has demon­strated several benefits, including smaller incisions, fewer intraoperative bleeding events, decreased adhesion for­mation, earlier recovery of bowel function, less postop­erative pain, shorter hospital stay, shorter recovery time, faster return to full activity, and low rates of surgical site infection and intra-abdominal con­tamination (18–20). However, some researchers have different opinions with respect to the significant difference between open and laparoscopic adhesiolysis (21). Laparoscopic access is safer in cases of uncomplicated adhesive intestinal obstruction (9, 22). However, the development of minimally invasive surgery techniques has led to greater need for surgical technology and equipment. Single-incision laparoscopic surgery (SILS), especially transumbilical surgery, has recently been introduced as a minimally invasive option for improving cosmetic outcomes and decreasing the risk of morbidity (23). Moreover, a correlational study revealed the surgical outcomes of single-incision laparoscopic surgery (SILS) were better than those of conventional multiport laparoscopic surgery in terms of the time to oral intake, intervention time, blood loss volume and length of hospital stay (24). However, single-incision laparoscopic surgery (SILS) for SBO is still not widely accepted because of the limited space for manoeuvrability of the instruments and the interference between the instruments, increasing the difficulty of the operation (25, 26).

In recent years, With the rapid development of laparoscopy and endoscopy cooperative surgery (LECS), endoscopy is increasingly widely used in surgery. The flexible endoscope has a flexible tip to allow observation from a different angle and can reach distant surgical sites. Moreover, with the implementation and development of precision surgery, faster and more accurate surgical resection is needed to reduce the risk of repeatedly touching the intestine and searching for adhesion areas, increasing the risk of adhesion formation. However, similar to NOTES, spatial orientation is still a major challenge in single-port laparoscopic or endoscopic surgery. Therefore, exploring a localization method to reduce the incidence of intraoperative irritation to the mucosa is critical to reduce the risk of intestinal adhesion recurrence.

Electromagnetic navigation technology has been preferred by clinicians in recent years because of its low risk of radiation exposure and accurate real-time localization. Moreover, electromagnetic navigation has been shown to be more accurate than optical navigation while avoiding the line-of-sight limitation inherent to optical systems (27). Lesion identification is faster with electromagnetic navigation. While a variety of technologies can be used for tracking in image-guided surgical navigation, they are not well suited for use in the moving anatomy of the intraperitoneal compartment (28). On the basis of previous research, we aimed to evaluate the feasibility and safety of dual-channel electromagnetic navigation technology in locating the target area in abdominal adhesions animal model.

The initial treatments for SBO include bowel rest, fluid repletion and placement of a nasogastric tube. Suter et al. reported that a bowel diameter exceeding 4 cm was associated with an increased rate of conversion (29). Bowel distension and the possibility of iatrogenic injuries are challenges associated with laparoscopic adhesiolysis (30). Related studies have shown that long nasal tubes are essential in terms of decompression of the intestinal contents, assessment of the stenotic lesion and creation of an excellent surgical field (31). During the early stage of model creation, we explored different doses of chemical agents to induce abdominal adhesions. 0.2% NaClO was used to induce the formation of fibrotic adhesions between the visceral layers of the peritoneum according to Hsu et al.'s research, and dose-dependent increase on the severity of multifocal adhesions 7 days after NaClO injection was observed in pigs. In our study, a gastric tube was placed before surgery for gastrointestinal decompression,. Only one case had bowel distension, which affected the visibility of the operative field. Two cases sustained minor iatrogenic intestinal injuries, which were not treated endoscopically. The peritoneum and operating space were clearly visible in other cases. Some organs, such as the liver, gallbladder, spleen, and diaphragm, were clearly identified. The abdominal wall structures were clearly visualized using a flipping endoscope. In our experiment, the first electromagnetic wire was delivered to the small intestine through a gastric tube to locate the target area. Then, the signal transmitter was activated, and the point on the abdominal surface closest to the first wire was identified using the second wire for skin incision. Following skin incision, the endoscope with a new electromagnetic wire was inserted into the abdominal cavity via a trocar and gradually approached the first electromagnetic wire according to the electromagnetic signal. We explored the abdominal cavity approximately 5 cm from the closest distance between the two wires and successfully found and cut off the adhesion bands. Our results showed that the localization time on the surface was significantly shorter than that in the abdominal cavity. The time of electromagnetic location accounted for 21% (16%–27%) of the total operation time.

During the operation, one case experienced intraoperative bleeding because the small mesenteric artery was damaged and endoscopic haemostasis failed. We failed to identify any bleeding vessels by exploring the omentum and intestine with endoscopic biopsy forceps, which reflects the limited ability of the flexible endoscope in moving and pulling tissues and organs. Another case of intraoperative bleeding was successfully resolved via endoscopic electrocoagulation. Furthermore, as surgeons become more experienced in performing the operation, the operation time gradually decreases. In our study, the reason why the forward distance of the endoscopy is longer than that shown by the navigation is that the flexible endoscopy could form curvature in the abdominal cavity. But this kind of situation could be improved between the near objections and within a small space. In this study, the endoscopic biopsy channel can accommodate the endoscope and all its accessories because they are placed in the same axial direction as the endoscopic body and there is barely any tension. Flexible endoscopy adhesiolysis is suitable for simple abdominal adhesion and unsuitable for complex intestinal adhesion. In addition to the defects of the device, most endoscopists are gastroenterologists, who do not have a deep understanding of the anatomy of the abdominal cavity. In our study, the detection of positive cultures without accompanying clinical infection suggests probable intraoperative contamination that was effectively contained. No cases progressed to abscess or systemic infection, implying competent local immunity. Nevertheless, this occurrence reinforces the need for rigorous aseptic protocols in future procedural refinements and clinical adaptation. The fidelity of electromagnetic navigation can be compromised by factors such as instrumental interference, bowel motility, and tissue displacement from pneumoperitoneum. Acknowledging these influences allows for a more balanced evaluation of the technique's present robustness. Consequently, mitigating these effects is paramount and is delineated as a priority for subsequent system optimization. In the future, wider clinical application will require the optimization of instruments. In the experiment, the postoperative condition of seven animals was good, and the animals returned to a normal diet after 24 h, with no apparent operational complications. Although the sample size in our study was small and the model was relatively simple, we explored the application of flexible endoscopy adhesiolysis in abdominal adhesions model and developed a new electromagnetic navigation technique for single-port endoscopic surgery for the first time. Although the induced multifocal intraperitoneal adhesions in the animal model did not result in complete bowel obstruction, however, in human cases, the precise location of intestinal obstruction can be identified through imaging modalities, and the use of contrast agents combined with ambulation may facilitate the long catheter to reach the obstruction site more quickly, we have proved the safety of this technique. Future investigations employing more severe adhesion models or those conducted within a phased clinical translation framework are warranted to comprehensively evaluate the technique's efficacy and safety in complex surgical scenarios. However, we acknowledge the limitations of using survival as a primary endpoint in this study. The absence of a sham-operated control group prevents a direct comparison of survival outcomes, limiting the interpretation of this metric. Therefore, the more meaningful data from this feasibility study lie in the technical performance of the procedure. The high rate of successful adhesiolysis (87.5%), the relative precision of electromagnetic navigation (achieving a median proximity indicator of 3–5 mm intra-abdominally), and the detailed account of adverse events such as bleeding and intestinal injury provide a more robust assessment of the technique's potential and current challenges. We will acknowledge that while the inter-sensor proximity was effective for procedural guidance in this model, absolute positional accuracy of the electromagnetic navigation system relative to anatomical structures was not formally quantified in this *in vivo* feasibility study due to organ movement and the lack of a static fiducial reference. These technical outcomes are critical for guiding future iterations and improvements of the navigation system and endoscopic tools. The translational constraints arising from differences in adhesion biology—notably, the lack of prior surgeries, divergent vascular patterns, and the less complex nature of adhesions in our model compared to the multifocal, chronic adhesions seen in human ASBO. This comparison cautions against the over-extrapolation of our results, while constructively outlining the necessary steps—such as validation in more complex adhesion models and phased clinical trials—required to bridge this translational gap.

Hereafter, we will continue to explore and improve the navigation system to be compatible with the body in larger-scale experiments in the future. Therefore, owing to continuous optimization, endoscopic interventions will become the preferred treatment option for patients suffering from gastrointestinal diseases.

## Conclusions

Single-port flexible endoscopy adhesiolysis for simple abdominal adhesions is safe and feasible. Moreover, dual-channel electromagnetic navigation technology for single-port endoscopic surgery is feasible and safe. It is relatively easy to locate the target area. Hence, the results of our experiment can serve as basis for spatial localization and flexible endoscopy in larger-scale experiments in the future.

## Data Availability

The original contributions presented in the study are included in the article/Supplementary Material, further inquiries can be directed to the corresponding authors.
